# Canine Scent Detection in Lung Cancer Screening

**DOI:** 10.7759/cureus.38877

**Published:** 2023-05-11

**Authors:** Megan Kudlak, Mohammed Mohammed Ali, Sarah Whitlow Kirk, Noah Medalsy, Heather Yoder, Harleen Bhullar

**Affiliations:** 1 Alumni, American University of the Caribbean School of Medicine, Cupecoy, SXM; 2 Medicine, American University of the Caribbean School of Medicine, Cupecoy, SXM

**Keywords:** cancer screening, canines in medicine, canine scent detection, medical canines, lung cancer screening, lung cancer

## Abstract

Canines historically have been proven to have great benefits in human medicine. They have a unique ability to detect volatile organic compounds, or VOCs, in several different diseases, which allows them to work efficiently as a medical alert dog or detect the presence of certain diseases in human samples. Early studies have shown efficiency in the ability of canines to detect malignant cells from primary lung tumors in the fluid and breath samples of patients. Lung cancer is the third most common cancer and is the number one cause of cancer-related deaths in the United States. Because of its commonality, The U.S. Preventive Services Task Force developed guidelines for screening high-risk individuals, which includes low-dose CT with proven efficacy. Although effective, it comes with limitations, including increased cost, concern for radiation exposure, and low compliance amongst those who are eligible for screening. Other screening methods have been studied to overcome these deficiencies, including the use of canines trained in medical scent detection. Medical scent canines may prove to be an efficient alternative form of screening to the traditional use of low-dose CT and may be a viable non-imaging screening alternative.

## Introduction and background

Canines and their powerful olfactory system have proven to be unique and valuable tools for many lines of work and are starting to be integrated into the medical field. They are known for their ease of handling and training due to their highly effective and sensitive olfactory system. Over the past several years, more research has been done to evaluate the canine's ability to detect infectious and non-infectious disease processes. Different diseases can either create or produce a higher ratio of volatile organic compounds, or VOCs [1]. These compounds can be present in breath, blood, urine, and sweat. Several diseases, such as cancer, cystic fibrosis, and asthma, can create or alter these VOCs, which then can be used as a biomarker for diagnostic purposes [2].

Different studies have shown that canines' olfactory abilities can be used to detect infectious and non-infectious diseases such as diabetes mellitus, cancer, malaria, and epilepsy [3]. For example, studies have proven that canines are able to use their olfactory system to detect hypoglycemia and hyperglycemia episodes in a patient with diabetes mellitus [4]. Canines have been proven to be reliable in detecting acute illnesses, such as those described, however, more recent research has been performed to evaluate the use of their scent skills in detecting malignancy in patients.

Canines' ability to detect cancer, more specifically lung cancer, continues to be studied today as it has the potential to bring a reliable, radiation-free, alternative form of detection. The canine’s olfactory system can help detect lung cancer in different tumor stages, including those in the earlier stages that are not always found in traditional screening methods. Canines have been studied to evaluate their ability in the detection of lung cancer either by sniffing a breath or blood sample. These samples contain VOCs produced by the malignancy, which the canines are able to detect through scent. Many studies have been done to determine the accuracy canines have in detecting these VOCs produced by lung malignancies [5-8]. In this review, we look at some of those prior studies that have shown this method of screening to be highly effective.

## Review

Canine ability to detect lung cancer

In 2006, a study was performed in California to find if dogs untrained in scent detection, would be capable of quickly learning how to detect patients with lung cancer. The study also included breath samples of patients with a diagnosis of breast cancer, however, those studies were done separately from the lung cancer samples. Researchers wanted to determine if the patient's age, smoking history, cancer stage, or recent meal consumption would influence the canines' diagnostic ability among cancer patients and healthy individuals. For this study, they used five household dogs between the ages of seven months and 18 months. As seen in Table 1, the dogs selected were three Labrador Retrievers and two Portuguese Water dogs. The researchers used criteria to select these canines based on suggestions by the American Kennel Club: dogs that are older than 6 months, are compliant with fundamental training, and have enthusiasm for sniffing. The training was structured into three phases. In the first phase, the dogs were trained to detect the sample positive either for adenocarcinoma or squamous cell carcinoma lung cancer, with a food item adjacent to the sample, out of four control samples. When the dog smelled the positive sample, a clicker was activated, a command to ‘sit’ was given as a way to train them to alert, and the dog was rewarded with food and praise. The second phase had the dogs detect the positive sample with a food item next to it and was rewarded with food and praise if they alerted to the correct sample. The third phase was similar to phase two, however, no food item was next to the correct sample. The canines were trained for 10 min in each session. After training these canines in scent detection, their ability to accurately detect breath samples from patients with a known diagnosis of lung malignancy was measured, as well as if they can differentiate breath samples of those patients from those of healthy individuals. Their sample size was 51 samples containing cells positive for lung cancer and 83 control samples from healthy individuals. The study suggests that canines were able to detect lung cancer from an “exhaled breath sample” with 99% sensitivity and 99% specificity in comparison to biopsy-confirmed samples [5]. The study's conclusion emphasized that it is possible to train household dogs to differentiate between breath samples of healthy individuals and those with lung cancer within a few weeks. The study also suggested that further research is necessary to explore the chemistry of exhaled breath to improve the accuracy of the results [5].

A study from 2011 in Germany evaluated the ability of canines to detect malignant cells from breath samples of patients with various types of lung cancers, including small cell carcinoma, adenocarcinoma, non-small cell carcinoma, and non-differentiated types. Four local owners volunteered their family dogs, between the ages of two and a half and three years old, to be trained by a professional handler for detection. The dogs were two females and two males, and their breeds were two German Shepherds, an Australian Shepherd, and a Labrador Retriever. The dogs wore a muzzle in training and were taught to signal the presence of a positive sample by lying down and touching their muzzles to the tube containing the sample. In order to test the dogs' ability to detect the positive sample, five separate retainers were used to hold the probes on the floor, with the rubber caps removed. The observers who were assessing the dogs' ability to detect cancer were kept blind to the position of the probes. One probe from a patient with confirmed lung cancer was used in each test, and its position was determined randomly by throwing a die. The individual who positioned the probes left the room before testing began, and a curtain was raised while the dogs were instructed to sniff the probes. A positive alert was recorded if the dog laid down in front of the positive tube with no hesitation. A total of 220 participants were enrolled to give breath samples; including 110 healthy individuals, 60 individuals with a prior diagnosis of lung cancer, and 50 individuals with a prior diagnosis of chronic obstructive pulmonary disease (COPD). A positive detection was recorded by two blinded study observers if the canine alerted on a sample without any hesitation. Although accuracy in the later stages of cancer was lower (Stage IV being 63%), there was a 100% accuracy in detecting Stage I cancer samples (Table 1). The overall results included a sensitivity of 71% and a specificity of 93% [6].

**Table 1:** Comparison of studies. Each study evaluating canine scent detection of lung cancer varies in the number of dogs, their breeds, and ages, but are similar in the modality of positive signals and rewards given to canines after a positive alert.

	McCulloch et al. (2006) [5]	Ehmann et al. (2011) [6]	Junqueira et al. (2019) [7]	Riedlova et al. (2022) [8]
Number of dogs	5	4	4	2
Breeds of dogs	3 Labrador Retrievers, 2 Portuguese Water Dogs	German Shepherd, Australian Shepherd, and Labrador Retriever	Beagles	Labrador Retriever, Australian Cattle Dog
Ages of dogs	7 months to 18 months	2.5-3 years	2 years	4 years, 5 years
Positive signal	Sitting in front of the sample	Lying down, touching muzzle to the tube containing the sample	Sitting in front of canister containing the sample	Lying down, sitting, scratching at the glass containing the sample
Reward	Food, praise	Unspecified	Food, clicker sound	Unspecified

A study in 2019 that took place in Florida evaluated four Beagles in their ability to detect malignant cells in blood samples from patients recently diagnosed with non-small cell lung cancer (NSCLC). These Beagles were not previously trained in scent detection of malignant cells but were selected due to the breeds' known olfactory ability and their ease of training. The dogs were trained using a clicker whenever they correctly identified a positive sample. Training took around 8 weeks. Three of the four dogs were able to correctly identify the positive samples; one dog was unmotivated during training and therefore was removed from the study. In the double-blind study phase, 30 samples positive for NSCLC were used, and 120 negative samples were used. The NSCLC samples were only used in this study due to how common it is and easy to treat if detected in early stages. Three of the four Beagles were able to correctly identify a positive sample. Although a small sample size, this study showed promising results (Table 1), with a sensitivity of 96.7% and specificity of 97.5% [7].

A 2022 study wanted to evaluate a canine's ability to detect lung malignancy in samples through a series of unblinded, single-blinded, and double-blinded tests. Two dogs were used from the Czech Center for Signal Animals. One dog was a 4-year-old Labrador Retriever and the other dog was a 5-year-old Australian Cattle Dog. The canines were trained in four phases. In the first phase, dogs were acquainted with the environment and materials. In the second phase, positive samples were introduced and in the third phase, negative samples were mixed in with the positive samples. Finally, in the fourth phase, blinded samples were given. The samples were presented on a steel plate with four holes housing work glasses with smell adsorbers inside. The smell adsorbers were replaced every training session and for each dog. The dogs sniffed all samples, stopped at the positive sample, and signaled to that sample in their own unique way (lying down, sitting, scratching). If no positive samples were present, the dog would return to the owner. The canines were rewarded with each correct marking. The positive samples used in the experiment consisted of 115 serum or breath samples from patients with either adenocarcinoma (30.4%), small cell carcinoma (33.9%), or squamous cell carcinoma (35.7%), all at various stages before they received chemotherapy or surgery. The control group consisted of 101 serum or breath samples from healthy people or those with other lung disorders who have not been previously diagnosed with any form of lung cancer. The patients were Indo-European men and women from the Czech Republic. As seen in Table 1, in the unblinded test, the Labrador Retriever had a sensitivity of 91% and a specificity of 92% while the Australian Cattle Dog had a sensitivity of 89% and a specificity of 81%. In the single-blinded studies, the Labrador Retriever had a sensitivity of 71%, and the Australian Cattle Dog had a sensitivity of 90%. For the double-blinded test, the results were split into two categories: serum and breath samples. For the Labrador Retriever, a sensitivity of 69% and a specificity of 67% was recorded for the serums test, whereas for the breath sample, a sensitivity of 62% and a specificity of 71% were recorded. The Australian Cattle Dog had a recorded sensitivity of 62% and specificity of 97% for the serums test, in comparison to the breath test, which resulted in a sensitivity of 75% and specificity of 90%. Overall, the Labrador Retriever had an accuracy of 68% and the Australian Cattle Dog had an accuracy of 83% [8].

Benefits of using canines

While the results from these studies are promising, we must further evaluate why alternative forms of screening, such as canine detection, should be researched more. Lung cancer is the leading cause of cancer-related death and the third most common form of cancer in the United States. In 2022, an estimated 236,740 cases of lung cancer were diagnosed, as well as an estimated 130,180 deaths from lung cancer [9]. Current guidelines from the United States Preventive Services Task Force recommend annual screening with Low-Dose Computed Tomography (LDCT) for all adults ages 50-80 years old who are at high risk of lung cancer due to smoking, those with a 20-pack-year history, who are currently smoking, or quit smoking within the last 15 years [10]. While screening for lung cancer provides early identification of a malignant process, there are issues to consider with the traditional screening method.

Unfortunately, lung cancer screening has been poorly accepted by the United States population most at risk. In 2022, only an estimated 5.8% of the eligible population in the United States was screened for lung cancer [11]. Factors including accessibility in underserved and rural areas, patients' understanding of the process, and screening-related anxiety, have been further evaluated through surveys in order to fully understand these low rates of lung cancer screening compliance. Out-of-pocket costs vary state-to-state, however, the out-of-pocket price patients have to pay for an LDCT can range from $100 to $500 per scan [12-13]. A survey conducted amongst high-risk patients reveals that while many are receptive to undergoing screening, willingness to participate in the traditional screening dropped by 50% due to cost [14]. Other potential concerns of lung cancer screening include a high false positivity rate. Looking at prior research, there can be up to a three-fold increase associated with the use of LDCT scans when compared to other methods, such as standard radiographic scans [15]. Although traditional screening with LDCT has detected several malignancies, the increased false positive rate can potentially lead to unnecessary tests and interventional procedures to rule out a malignancy, thus increasing the risk of medical complications ranging from infection to radiation-induced cancer to death [16-17].

Utilizing canine sniffing for lung cancer detection could be a potential solution for patient concerns regarding traditional screening, as it has been proven to be inexpensive and uses a less invasive approach that requires no radiation. Compared to the sensitivity and specificity of LDCT to detect lung cancer, which is 88.9% and 92.6%, respectively, medical scent canines are comparable in effectiveness when detecting any stage of lung cancer, as was presented with the prior data discussed [18] (Table 2).

**Table 2:** Low-dose CT vs. medical scent canines. Medical scent canines are comparable in sensitivity, have higher specificity, and can potentially cost less than the traditionally used low-dose CT in lung cancer screening. *This price estimate comes from prior use of medical scent canines used in research for detecting coronavirus disease 2019 (COVID-19).

	Low-dose CT [18-19]	Medical scent canines [5-8, 20]
Sensitivity	88.9%	71%-99%
Sensitivity	92.6%	93%-99%
Cost to run	$3,074 per person	~$79 per day*

Additionally, the use of medical scent canines is less of a financial burden than that of a CT scanner. The cost for programs to run a LDCT scan is an estimated $3,074 per patient, and the price of purchasing a CT scanner can range from $69,900 to $249,500 [19]. This is excluding yearly maintenance, staff salaries, and insurance cost to patients. In contrast, canines cost only a fraction of what a CT scanner would. To estimate the cost that canines would be in cancer screening, we can look at the estimated daily cost of scent detection dogs used for coronavirus disease 2019 diagnosis that was evaluated in a 2022 study. This was estimated to be $79 USD, equating to around an annual cost of $20,540 if the dogs were used five days a week [20]. While further evaluation of detailed price per screening test utilizing canines is still needed, the total price of having a canine in service for five years is anticipated to be hundreds of thousands of dollars less than the operation of the CT machine as seen in Table 2.

Limitations of medical scent canines

Canine scent detection has a promising future, but there are some inconsistencies when it comes to using them for screening. There is no generalized consensus on when a canine has completed its training. Some studies consider training to be completed when a certain time frame has been met, whereas other studies have a benchmark goal that needs to be met for training to be considered complete. Others consider training complete when a benchmark goal has been met during each phase of training; not allowing the canine to continue unless it has properly met the benchmark required regardless of the time it takes [5]. This brings into question canines' ability to detect lung cancer due to the lack of standardized training or even the lack of criteria needed to consider these canines eligible for work. Unlike LDCT, canine scent detection can be negatively impacted by environmental factors. Testing conditions that are unwarily inappropriate for canines like high humidity and elevated ambient temperatures without being able to acclimate were found to be detrimental to the dog’s sense of smell [21]. A canine's homeostatic compensatory response to increase heat from its environment is to pant in order to cool down [22]. This, in turn, decreases and compromises the olfactory senses in canines. Another confounding factor, in cases where breath samples are being used, is the presence of smokers' breath from tobacco. Canines aiming to detect lung cancer in patients who regularly smoke will be hindered due to the masking smell of smoke [23]. This can also be said for food odors and several drug metabolites for cancer therapy [24]. These factors must be addressed when undergoing canine scent detection, as testing must be in a controlled environment, and patients must be educated on cessation of smoking prior to giving a breath sample.

## Conclusions

While it is well established that LDCT scanning is the gold standard for lung cancer detection, low patient compliance has doctors questioning if other methods of screening can be introduced. Canines could provide substantial benefits in the medical field, specifically in the early detection of lung malignancy. The benefits of canine scent detection in the identification of lung cancer include cost effectiveness, decreased test-related patient anxiety, decreased repeat radiation exposure, increased patient accessibility, and possibly increased patient compliance to screening. Through more research, further analysis of the sensitivity and specificity can be performed, however, there are many other aspects of canines in lung cancer screening that must be evaluated. Considerations that still need to be evaluated include the quality of canine training, cost per canine test, length of service for each canine, and how many canines are needed per screening program based on the community population. The methods to ensure continuity of training across various dog breeds need to be firmly established with a defined benchmark indicating the dog has received the necessary training to detect malignancies accurately. A total cost per canine test must be accurately calculated to include the cost of dog training and materials used and to determine how cost-effective canine screening would be in comparison to LDCT scans. Another consideration is whether canine screening might be covered by insurance and/or Medicaid/Medicare. Non-CT methods, including canine screening, are resources that need to be explored more, and further research needs to be performed to test the effectiveness of these non-imaging-based approaches. Other competing non-imaging options, such as electronic noses, must also be considered and have their efficiency measured against scent canines trained to detect lung malignancy. 

Canine scent detection has a promising future in detecting early cases of lung cancer, as the prior studies discussed showed high accuracy rates. Although these results look bright, only a few canines were used per study as well as limited sample sizes for them to work with. More research must be performed to see what training methods develop the most accurate results and form a protocol for training future canines in lung cancer detection. LDCT scanning continues to be the primary method for lung cancer screening at this time, but other methods such as canine detection should undergo further research to present alternative screening options to patients.
